# RNA-binding protein gene NOP58 exhibits crucial prognostic and therapeutic value in Ewing sarcoma

**DOI:** 10.1186/s41065-025-00440-5

**Published:** 2025-05-14

**Authors:** Yannan Geng, Lu Yang, Rui Shao, Tiantong Xu, Lilong Zhang

**Affiliations:** 1 Department of the Sixth Spinal Surgery, Tianjin Union Medical Center, The First Affiliated Hospital of Nankai University, No. 190 Jieyuan Road, Hongqiao District, Tianjin, 300121 China; 2 The Third Department of Breast Cancer, Tianjin Medical University Cancer Institute and Hospital, Tianjin, 300060 China

**Keywords:** NOP58, Ewing sarcoma, RNA-binding protein, Prognosis, Cell growth

## Abstract

**Background:**

Our aim was to identify crucial RNA-binding proteins (RBP) genes associated with Ewing sarcoma (EwS) in order to provide valuable insights into its mechanisms of tumorigenesis and to enhance therapeutic intervention.

**Results:**

Differential gene expression analysis identified candidate genes. Next, hub genes were generated by the results of protein-protein interaction (PPI) network, and univariate COX regression analysis. CIBERSORT was applied to analyze immune landscape. Furthermore, both in vitro and in vivo experiments were conducted to investigate the function of NOP58 in EwS.

**Results:**

A total of 179 RBP-related genes were significantly different in EwS tissues and normal controls. Among these, NOP58 ribonucleoprotein (NOP58) was considered as the hub gene, demonstrating significant prognostic value. Significantly, high NOP58 expression correlated with poor prognosis of EwS patients. Additionally, the levels of NOP58 were significantly up-regulated in EwS cells compared with human mesenchymal stem cells. Furthermore, knockdown of NOP58 notably inhibited the proliferation and migration of EwS cells. Moreover, NOP58 deficiency remarkably induced apoptosis and cell cycle arrest in EwS cells. In vivo studies on tumor-bearing mice demonstrated that NOP58 downregulation significantly inhibited tumor growth in EwS.

**Conclusion:**

Collectively, downregulation of NOP58 could inhibit the proliferation and migration of EwS cells in vitro and reduce murine xenograft tumor growth in vivo. These findings identified NOP58 as a promising regulator of EwS tumorigenesis, suggesting it may serve as a potential therapeutic target for EwS treatment.

**Supplementary Information:**

The online version contains supplementary material available at 10.1186/s41065-025-00440-5.

## Introduction

Ewing sarcoma, or Ewing’s sarcoma (EwS) is a small round cell sarcoma that forms in bones and soft tissues [1]. This includes various forms such as EwS of bone, peripheral primitive neuroectodermal tumor, extraosseous EwS, and Askin tumor (EwS of the chest wall) [2]. Dr. James Ewing was the first to discern this type of tumor as a distinct cancer [3]. EwS is characterized by its high aggressiveness and primary and is the second most common primary malignant bone tumor in children and adolescents [4]. According to 2024 cancer statistics report, Ewing tumor & related bone sarcomas has an incidence rate of 2.6 per million in children and 4.6 per million in adolescents in the United States, with 5-year survival rates of 81% and 68%, respectively [5]. Notably, EwS exhibits early metastatic potential, high relapse rates, and low 5-year survival [6]. Specifically, about 15–25% of the patients with EwS are diagnosed with metastases at presentation [7]. The relapse rate for surgically-resected EwS is as high as 90% in the absence of systemic chemotherapy [8]. Recently, the management of cancers primarily relies on a multimodal approach, integrating chemotherapy, surgical resection, and/or radiotherapy as the standard of care [9]. Until now, no molecularly targeted drugs or immunotherapies have yet received the Food and Drug Administration (FDA) approval for EwS; thus EwS is generally treated using a multimodal approach, including systemic chemotherapy, surgery and/or radiation [8]. Nevertheless, patient responses to treatment can vary largely, complicating the management of EwS [10]. A deeper understanding of EwS pathogenesis is crucial for improving prognosis and developing personalized treatment strategies.

RNA-binding proteins (RBPs) typically bind to mature mRNA sequences and are ubiquitously expressed. They are deeply conserved evolutionarily and considered to play central roles in gene regulation and the destiny of mRNAs [11], such as RNA splicing, cleavage, transport, maintenance of RNA stability and degradation, intracellular localization, editing and translation control [12]. RBPs are also vital players and coordinators in the maintenance of genome integrity [13]. Alterations in the expression or activity of RBPs can lead to various disorders or diseases including sarcomas [14, 15]. For instance, the RBPs LARP4A and LARP4B have the potential to enhance the growth and metastasis of sarcoma [16]. It is therefore not surprising that RBPs also play vital roles in EwS. For example, 29 RBP genes were identified as significantly prognostic-associated genes in EwS [17]. What is more, Ewing Sarcoma protein (EWS), a multifaceted RNA binding protein, is considered to modulate transcription, pre-mRNA splicing, and particularly, epigenetic remodeling during the tumorigenesis of EwS [17, 18]. The expression of the fusion protein EWSR1-FLI1 generated by chromosome translocation gives rise to more than 85% of EwS cases [1, 19]. Despite the significant role of RBPs in EwS, most research has focused on EWS or EWSR1. Studies on other EwS-related RBPs are limited, and further investigation in this area could enhance our understanding of the underlying mechanisms and contribute to the development of more effective therapeutic interventions.

## Materials and methods

### Data collection

In this study, we downloaded GSE68776, GSE45544, GSE17674, GSE17618, GSE17679 and GSE218758 datasets from GEO (Gene Expression Omnibus, https://www.ncbi.nlm.nih.gov/geo/). GSE68776 contains 32 EwS biopsy specimens and 33 normal samples; GSE45544 contains 14 EwS samples and 22 normal samples; GSE17674 contains 44 EwS samples and 18 normal samples; GSE17618 contains 44 EwS samples; GSE17679 contains 99 EwS samples and 18 normal samples; GSE218758 contains 19 EwS samples. https://dcc.icgc.org/.

All RBP genes were obtained from previous reports (Table S1) [17].

### Differential gene expression analysis

All subsequent statistical analyses were conducted using R software (version 4.2.1). Based on the limma [20] function package, the differentially expressed genes (DEGs) were screened by criteria of the absolute value of the logarithmically transformed fold change|log_2_FC| > 1 and false discovery rate (FDR) < 0.05.

### Pathway enrichment analysis

GO (Gene Ontology) enrichment analysis (including Biological Process (BP), Molecular Function (MF) and Cellular Component (CC)) and KEGG (Kyoto Encyclopedia of Genes and Genomes) pathway enrichment analysis were carried out on candidate genes by clusterProfiler package of R language [21, 22]. Significantly enriched GO terms and KEGG pathways were identified using a threshold of p.adjust < 0.05.

### Protein-protein interaction (PPI) network analysis

STRING [23] is a database for analyzing and predicting proteins’ functional linkages and their interactions. We used STRING (https://string-db.org/, version 11.0) to analyze the interaction between proteins [24–26]. Cytoscape (version 3.7.2) was used to visualize the PPI network [27]. Then we further screened the key genes in the PPI network by the cytoHubba plug-in in Cytoscape software based on the Maximum neighborhood component (MNC) algorithm.

### Gene set enrichment analysis (GSEA)

GSEA was conducted between these two groups using the R language function package and clusterProfiler (version 4.8.3) [21]. The significantly enriched pathways were screened by the standard of p.adjust < 0.05.

### Immune cell infiltration analysis

The relative proportions of 22 immune cell types in EwS samples in GSE17618 was calculated using CIBERSORT [28]. CIBERSORT employs the deconvolution algorithm to characterize the composition of infiltrating immune cells using the preset 547 barcode genes based on the gene expression matrix. The sum of all estimated proportions of immune cell types in each sample is equal to 1. Furthermore, xCell was utilized to determine the relative distribution of 36 different types of immune cells present across the two groups [29]. Additionally, the ESTIMATE function package was employed to estimate the infiltration levels of stromal and immune cells within tumor tissues, generating three scores: the stromal, immune, and estimate scores [30].

### Survival analysis

The overall survival rate of different groups based on the Kaplan-Meier method was estimated by survival package and survminer package (https://CRAN.R-project.org/package=survival). Then, log-rank was used to test the significance of the difference in survival rate between different groups. After that, we conducted a multivariate Cox regression analysis to determine whether NOP58 serves as an independent prognostic factor for survival in EwS patients. The ROC (receiver operating characteristic) curve was made by timeROC package to evaluate the predictive prognostic value of NOP58 [31].

### Cell lines culturing

One human mesenchymal stem cell line (hMSC) and three strains of human EwS cell lines: A673, RD-ES, and SK-N-MC were used in this study. The hMSC, RD-ES, and SK-N-MC cell lines were purchased from Shanghai Zhong Qiao Xin Zhou Biotechnology Co.,Ltd. (Shanghai, China), while A673 cells were purchased from Procell Life Science&Technology Co.,Ltd. (Wuhan, Hubei, China). The hMSC cells were cultured in specialized human mesenchymal stem cell culture medium ZQ-1320 (Zhongqiao Xinzhou). A673 cells were cultured in Dulbecco’s Modified Eagle Medium (DMEM) (C11995500BT, GIBCO (Grand Island Biological Company), Waltham, MA, US) supplemented with 10% fetal bovine serum (FBS) (FS401-02, TransGen Biotech Co., LTD, Beijing, China) and 1% Penicillin-Streptomycin (P/S). RD-ES cells were cultured in Roswell Park Memorial Institute medium (RPMI)-1640 (C11875500BT, GIBCO, Waltham, MA, US) supplemented with 10% FBS and 1% P/S. SK-N-MC cells were cultured in Minimum Essential Medium (MEM) (containing non-essential amino acids (NEAA), ZQ-1320, Zhongqiao Xinzhou) supplemented with 10% FBS and 1% P/S. All four types of cells were incubated at 37 °C in a culture incubator with 5% CO_2_ and saturated humidity.

The lentiviral empty vector (SORT-B19, BIOSETTIA INC., San Diego, CA, US) was transfected into SK-N-MC and A673 cells using Lipofectamine 3000 (L3000015, Invitrogen Corporation, Waltham, MA, US).

### Immunofluorescence (IF) staining assay

SK-N-MC and A673 cells underwent fixation using 4% paraformaldehyde, followed by permeabilization with 0.5% Triton X-100 and blocking with 5% BSA for 1 h. Subsequently, cells were probed with anti‐NOP58 primary antibody at 4 °C overnight, followed by incubation with Alexa Fluor 488‐labelled secondary antibody for 2 h. Following this, the nuclei were stained with DAPI reagent for 10 min. The immunofluorescence was eventually observed using an immunofluorescence microscope.

### Western blot

After protein extraction using Radioimmunoprecipitation assay (RIPA) lysis buffer and phenylmethylsulfonyl fluoride (PMSF) (R0010, Beijing Solarbio Science & Technology Co., Ltd., Beijing, China), protein concentrations were determined using the Bicinchoninic acid (BCA) protein concentration assay kit (PC0020, Solarbio). The western blot experiments were performed following established protocols as described previously [32]. Primary antibodies against NOP58 (ab155969, 1:2000 dilution, Abcam, Inc., Cambridge, UK) and β-actin (66009-1-Ig, 1:50000 dilution, Proteintech Group, Inc., Rosemont, IL, US) were used. Secondary antibodies, anti-rabbit IgG HRP-linked antibody for NOP58 (7075, 1:5000 dilution) and anti-mouse IgG HRP-linked antibody for β-actin (7076, 1:5000 dilution), were purchased from Cell Signaling Technology (CST, Danvers, MA, US). Finally, band intensities were semi-quantified using Image J software.

### Quantitative real-time PCR (qRT-PCR)

Total cellular RNA was isolated using TRNzol reagent (15596026, Invitrogen Corporation, Waltham, MA, US). A UV spectrophotometer was employed to assess the RNA’s purity and concentration. Complementary DNA (cDNA) was synthesized from the extracted RNA using TransScript^®^ IV One-Step gDNA Removal and cDNA Synthesis SuperMix (AW311-02, Transgen Biotech Co., LTD, Beijing, China). Subsequently, qRT-PCR was conducted with the PerfectStart^®^ Fast Green qPCR SuperMix (AQ611, Transgen) on a CFX96 Touch Real-Time PCR Detection System (Bio-Rad Laboratories, Inc., Hercules, CA, US), following the thermal profile: an initial denaturation at 95 °C for 60 s, followed by 40 cycles of denaturation at 95 °C for 5 s and annealing/extension at 55 °C for 15 s. GAPDH was utilized as the reference gene, and the primer sequences are listed in Table 1. Each reaction was performed in triplicate to determine the relative mRNA expression levels, which were quantified using the 2^−ΔΔCT^ method.

**Table 1:** Primer sequences of qRT-PCR

Target gene	Primer sequence	Tm (℃)
NOP58-F	CAGAAAGTTGGCGATAGGAA	55
NOP58-R	AACCATGACTGTAACATTGGGT	55
GAPDH-F	AGATCCCTCCAAAATCAAGTGG	55
GAPDH-R	GGCAGAGATGATGACCCTTTT	55

### CCK-8 assay

Cells were seeded in a 96-well plate and cultured for 24 h. Afterward, 100 µl of CCK-8 solution (CK04, Dojindo Molecular Technologies, Inc., Kumamoto, Japan) was added to each well, followed by incubation at 37 °C in a 5% CO_2_ incubator for 3 h. The optical density (OD) values of each well were measured at 450 nm wavelength using a microplate reader.

### Transwell migration assay

4 × 10^4^ cells suspended in serum-free medium were seeded into the upper chamber of the Transwell, and 600 µL of culture medium containing 10% FBS was added to the lower chamber. Cells were incubated for 24 h in a humidified incubator at 37 °C with 5% CO_2_. Following incubation, the cells were fixed with 4% paraformaldehyde for 10 min, and then stained with crystal violet for 15 min. Subsequently, the bottom surface of the Transwell membrane was air-dried, and the migratory cells were taken under a light microscope.

### Wound healing assay

Cells were seeded in a 6-well plate, and cultured for 24 h at 37 °C. When the cells formed a monolayer covering the bottom of the plate, a scratch was made in the center of each well using a 200 µL pipette tip. After washing the cells three times with PBS, the culture medium was replaced with serum-free medium, and cells were cultured for 0 and 16 h at 37 °C in a 5% CO_2_ incubator. Finally, images were captured under a light microscope. Image J software was used to measure wound distances. The migration distance of cells was measured according to the following formula: the wound distance between cells at 0 h minus the wound distance between these cells at 16 h.

### Flow cytometry for cell cycle analysis

For cell cycle analysis, a cell cycle detection kit (4087 S, CST, Danvers, MA, US) was used. Briefly, cells were suspended in ice-cold phosphate buffered saline (PBS), and then fixed in 75% ethanol at 4 °C overnight. Next, the cells were treated with 20 µg/ml RNase at 37 °C for 30 min, followed by staining with 50 µg/ml propidium iodide (PI) staining solution at room temperature in the dark for 30 min. The samples were analyzed using a BD FACSC Canto II flow cytometer (Becton, Dickinson and Company, Franklin Lakes, NJ, US).

For cell apoptosis analysis, an apoptosis detection kit (556547, BD) was applied. Briefly, cells were resuspended in 300 µL of 1× Binding buffer and stained with 5 µL of Annexin V-fluorescein isothiocyanate (FITC) in the dark for 15 min, following by staining with 5 µL of PI for 5 min before detection. The results were analyzed using a BD FACSC Canto II flow cytometer.

### Terminal deoxynucleotidyl transferase-mediated dUTP nick end labeling (TUNEL) assay

Cell apoptosis was assessed using the Click-iT™ Plus TUNEL Assay Kit (C10617, Invitrogen) following the manufacturer’s instructions. Images were captured under a fluorescence microscope.

### In vivo tumor growth assay

The animal experiments conducted in this study received full approval from the Medical Ethics Committee of Tianjin Union Medical Center (Ref No. 2024-B05). All the animal experiments were complied with the guidelines of the National Institutes of Health Guidelines for Care and Use of Laboratory Animals and were performed in accordance with the ARRIVE guidelines.

Female NSG triple-immunodeficient mice at 5 weeks of age were obtained from SPF (Beijing) BIOTECHNOLOGY Co., Ltd. (Beijing, China) for xenograft tumor modeling. A total of 5 × 10^6^ sh Ctrl-transfected or NOP58-knockdown SK-N-MC cells (in 100 µL) were subcutaneously inoculated into the mice, with each group consisting of 5 mice. The tumor size was measured every three days after tumor formation. The tumor volume was calculated with the formula: tumor volume = length × width × width × 0.5. After 15 days, all mice were euthanized by cervical dislocation under anesthesia.

### Statistical analyses

The Wilcoxon rank sum test was employed to calculate confidence intervals (CIs) for various groups and to compare differences in gene expression and the infiltration of immune cells between different groups [33, 34]; a narrower CI indicates a more precise estimate of effect size, whereas a wider CI reflects a less precise estimate, and A *p* < 0.05 was considered statistically significant. Spearman’s rank correlation analysis was performed using the “cor” function of R language, with Fisher’s transformation method applied to compute CIs. When *p* < 0.05, the difference was considered statistically significant.

The experimental data were presented as mean ± standard deviation (Mean ± SD). All data were processed and statistically analyzed using GraphPad Prism 6. Comparisons between the means of two groups of samples were conducted using t-tests, while comparisons between three or more groups were performed using one way analysis of variance (ANOVA) tests. A p-value of < 0.05 was considered statistically significant for indicating differences.

## Results

### Abnormally expressed RBP genes in EwS samples and their potential functions

**Fig. 1:**
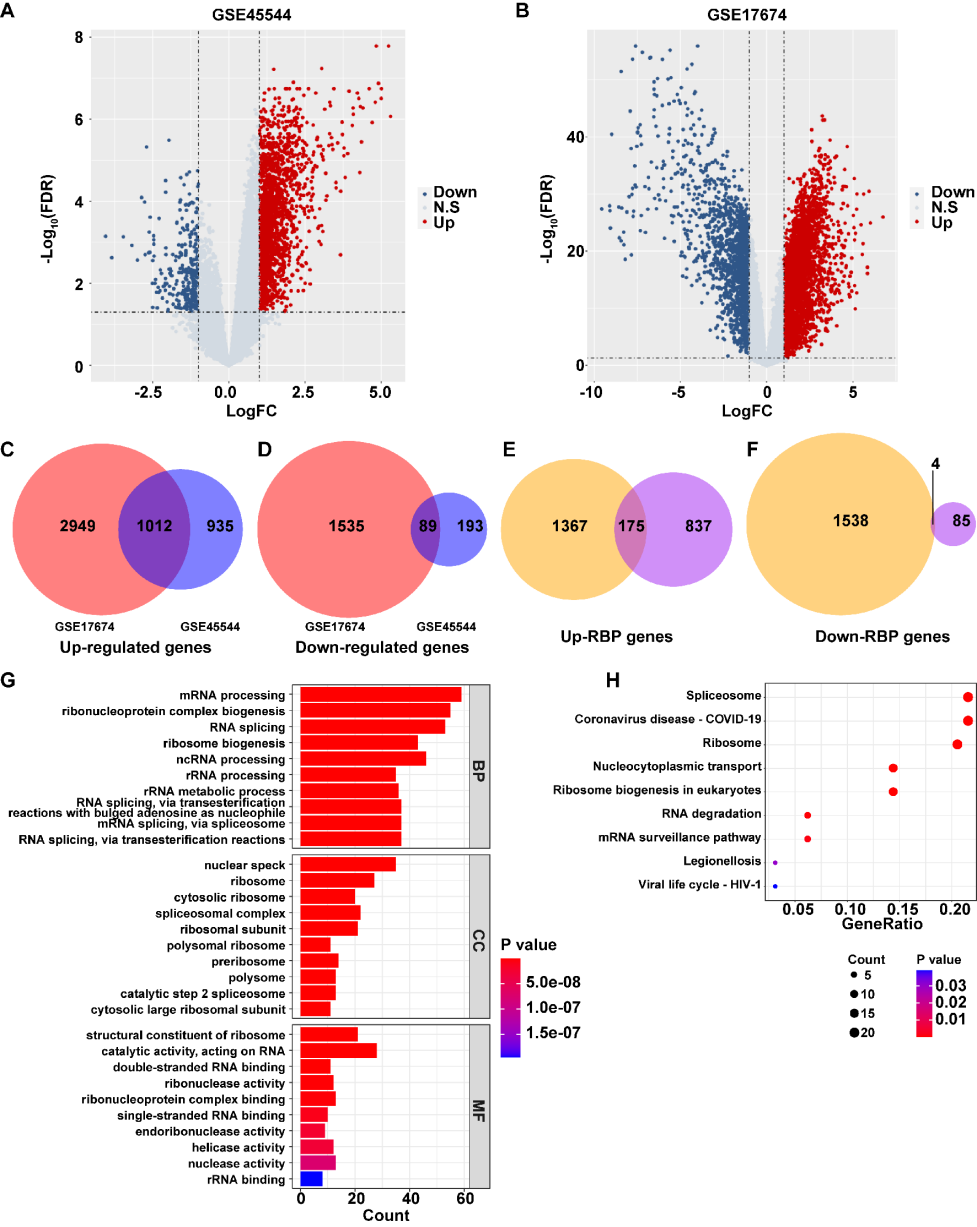
Differential gene expression analysis and DEGs’ potential functions. (**A**). DEGs between EwS samples and normal samples in GSE45544. (**B**). DEGs between EwS samples and normal samples in GSE17674. (**C**-**D**). Upregulated and downregulated genes of the intersection of DEGs from the two datasets. (**E**-**F**). Intersection between RBP genes and upregulated and downregulated genes showed in Figure C. and D. (**G**). The top 10 significantly enriched BP, CC and MF entries of GO terms. (**H**). Nine KEGG pathways with the most significant enrichment

Differential gene expression analysis was performed to identify DEGs between EwS and normal samples in GSE45544 and GSE17674 datasets, respectively. In the GSE45544 dataset, a total of 2229 DEGs were identified in EwS samples compared to normal samples, including 1947 up-regulated genes and 282 down-regulated genes (Fig. 1A). In the GSE17674 dataset, a total of 5585 DEGs were identified in EwS samples compared to normal samples, including 3961 up-regulated genes and 1624 down-regulated genes (Fig. 1B). The Venn diagram software was utilized to analyze common DEGs between GSE45544 and GSE17674 datasets. The results showed 1012 common up-regulated genes and 89 common down-regulated genes in two datasets (Fig. 1C and D). Next, these common genes were then intersected with RBP genes (Table S1 [17]), resulting in the identification of 179 common RBP genes (Fig. 1E and F, Table S2). Compared to normal samples, 175 RBP genes were found to be up-regulated and 4 RBP genes down-regulated in EwS samples in these two datasets (Fig. 1E and F, Table S2).

GO and KEGG enrichment analysis were performed on the above 179 candidate genes. 325 BP, 95 MF, and 77 CC entries were found to be significantly enriched (P value < 0.05). Meanwhile, 9 significantly enriched KEGG pathways were obtained (Table S3). The top 10 significantly enriched BP, CC and MF terms, along with nine significantly enriched KEGG pathways, were shown in Fig. 1G and H.

### Crucial RBP gene NOP58 was associated with the genesis of EwS

**Fig. 2:**
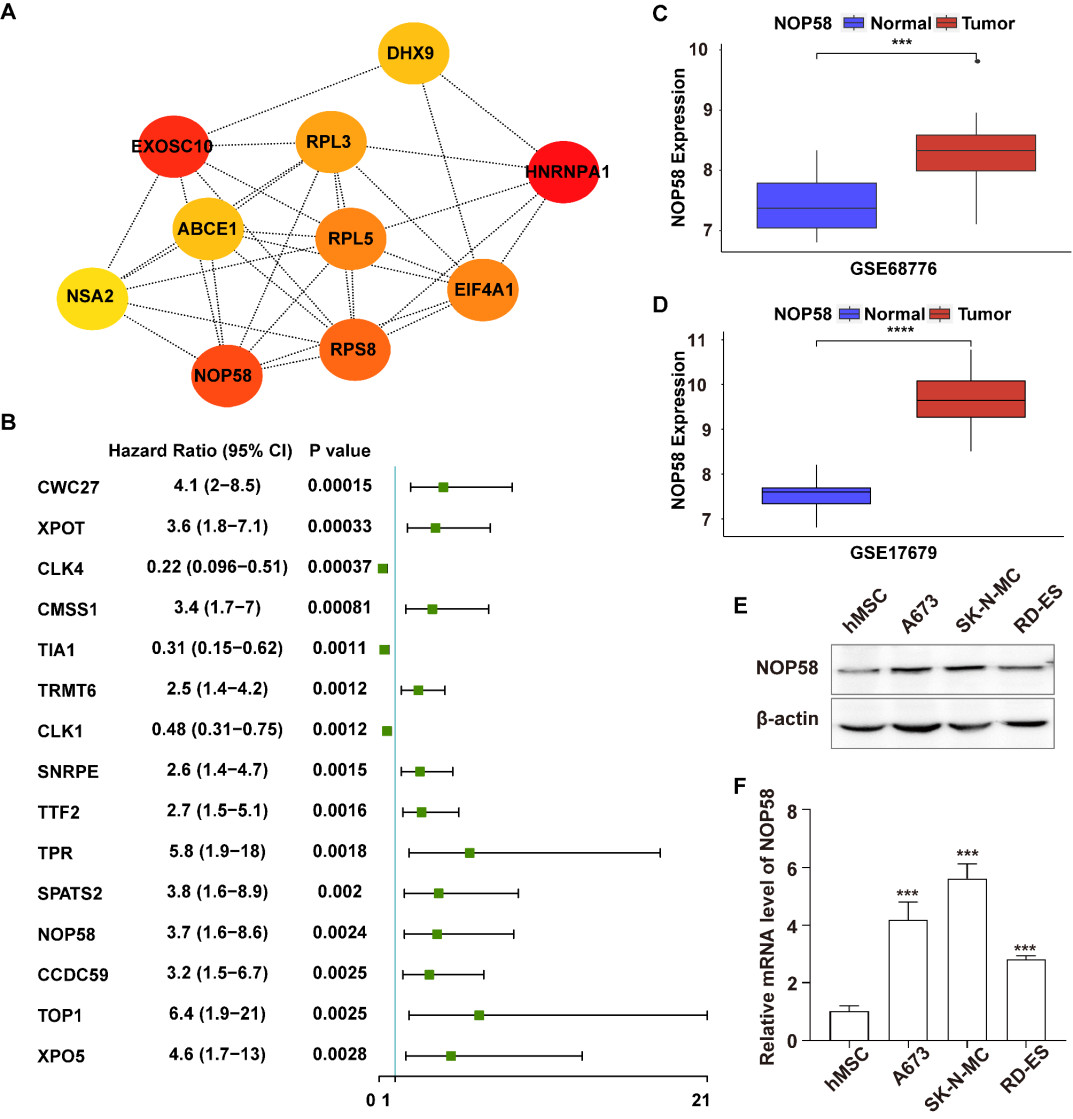
Crucial RBP gene NOP58 in the progression of EwS. (**A**). The PPI network of top 10 genes in the 179 candidate genes. (**B**). The top 15 genes screened out by P value of univariate COX regression analysis on 179 candidate genes. (**C**). The NOP58’s expression of tumor group and normal group in GSE68776. (**D**). The NOP58’s expression of tumor group and normal group in GSE17679. (**E**) Western blot and (**F**) qRT-PCR analysis of NOP58 expression in normal and EwS cell lines

To develop potential crucial genes for further investigation, STRING database was conducted to analyze PPI networks, with minimum required interaction score > 0.4 as the threshold for screening interaction pairs. The PPI network was shown in Figure S1 after visualized by Cytoscape software. Subsequently, we analyzed the topological structure of the entire PPI network by Cytoscape software, and ranked the importance of each node in the network according to the Maximum Neighborhood Component (MNC) algorithm [35]. A darker color indicates a higher score, signifying that the node is more important. The top 10 important genes were heterogeneous nuclear ribonucleoprotein A1 (HNRNPA1), exosome component 10 (EXOSC10), NOP58 ribonucleoprotein (NOP58), ribosomal protein S8 (RPS8), eukaryotic translation initiation factor 4A1 (EIF4A1), ribosomal protein L5 (RPL5), ribosomal protein L3 (RPL3), ATP binding cassette subfamily E member 1 (ABCE1), DExH-box helicase 9 (DHX9) and NSA2 ribosome biogenesis factor (NSA2) (Fig. 2A).

Meanwhile, utilizing the data in the GSE17674 cohort, univariate COX regression analysis was performed on 179 candidate genes to identify the prognosis-related genes, and each gene’s HR (Hazard ratio) was calculated. The top 15 genes were screened out based on their P values (Fig. 2B). By intersecting the top 15 prognosis-related genes (Fig. 2B) with top 10 important genes identified through the MNC algorithm (Fig. 2A), NOP58 was finally identified as a crucial RBP gene with prognostic value in EwS. The expression of NOP58 between EwS samples and normal samples was independently analyzed in the GSE68776 and GSE17679 datasets, and the results showed that the expression of NOP58 was significantly upregulated in EwS samples compared to normal samples (Fig. 2C and D). To validate the expression of NOP58 in EwS, we conducted western blot (Fig. 2E) and qRT-PCR (Fig. 2F) experiments using a strain of hMSC and three strains of human EwS cell lines. The results showed that compared to hMSC cells, NOP58 levels were significantly elevated in A673, RD-ES, and SK-N-MC cells (Fig. 2E and F), which were consistent with the findings from our bioinformatics analysis.

### Pan-cancer analysis of NOP58

**Fig. 3:**
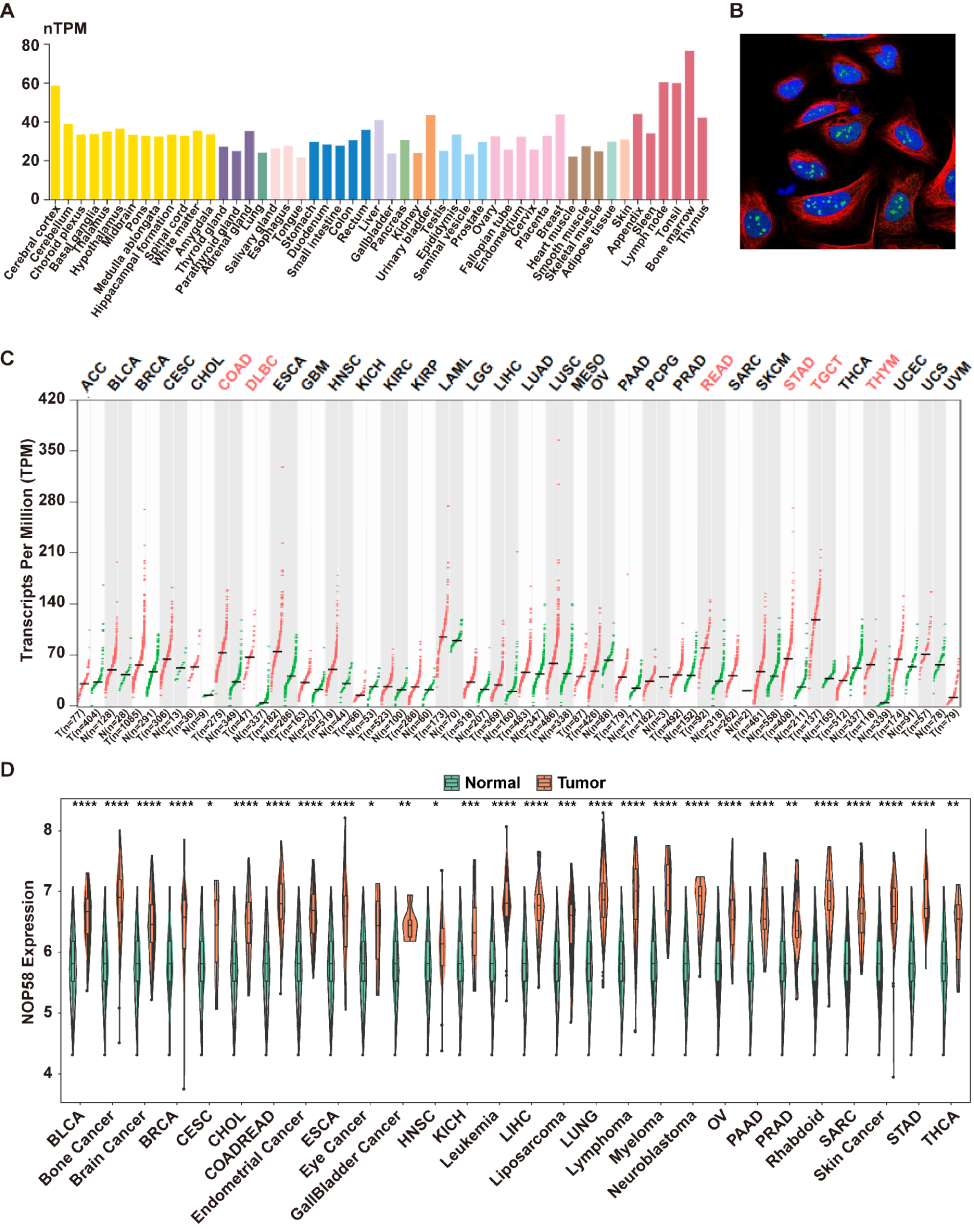
Pan-cancer analysis of NOP58. (**A**). The expression of NOP58 in various normal tissues in HPA database. (**B**). Subcellular localization of NOP58 in osteosarcoma cell line U2OS in HPA database. (**C**). The expression of NOP58 in different tissues in GEPIA database. (**D**). NOP58 expression in different cell lines in CCLE database. * meant p was smaller or equaled 0.05, ** meant p was smaller or equaled 0.01, *** meant p was smaller or equaled 0.001, **** meant p was smaller or equaled 0.0001

Considering the important role of NOP58 in EwS, we further investigated its expression across various cancer-related databases. In the Human Protein Atlas (HPA) database, we observed that NOP58 was expressed in different tissues and exhibited less tissue specificity in normal tissues. The expression levels in several muscle tissues were relatively low (Fig. 3A). In addition, the subcellular localization results for NOP58 in osteosarcoma cell lines, as reported in the HPA database, indicated that in the U2OS cell line, NOP58 protein was mainly localized in the nucleolus fibrillar center (Fig. 3B). Pan-cancer analysis of the NOP58 gene was conducted using the GEPIA (Gene Expression Profiling Interactive Analysis, https://gepia.cancer-pku.cn/) database, and the results showed that NOP58 was significantly upregulated in tumor tissues of COAD (colon adenocarcinoma), DLBCL (diffuse large B cell lymphoma), READ (rectum adenocarcinoma), STAD (stomach adenocarcinoma), TGCT (Testicular Germ Cell Tumors) and THYM (Thymoma) compared to normal controls (Fig. 3C). Furthermore, we utilized the CCLE (Cancer Cell Line Encyclopedia) database (https://sites.broadinstitute.org/ccle) to analyze NOP58 expression in various cancer cell lines, which demonstrated that its expression was significantly up-regulated in multiple cancer cell lines compared with normal controls (Fig. 3D).

### Immune landscape related to NOP58 in EwS

**Fig. 4:**
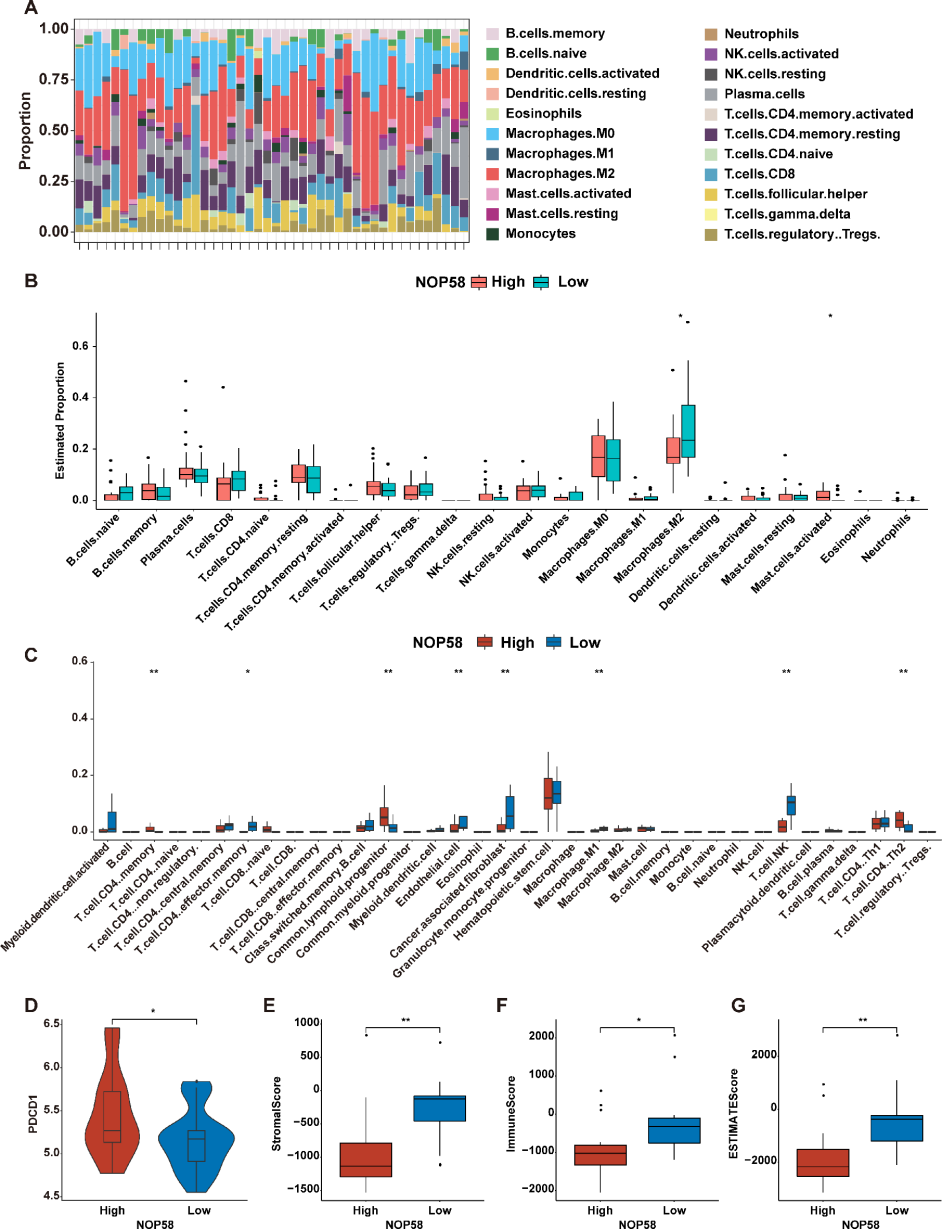
The results of immune infiltration analysis. (**A**). The relative content of 22 kinds of immune infiltrating cells in NOP58 high and low expression groups in GSE17618. (**B**). The CIBERSORT algorithm was employed to evaluate the immune infiltration of 22 kinds of immune cells among the samples in NOP58 high and low expression groups in the GSE17618 dataset. (**C**). The xCell algorithm was conducted to assess the immune infiltration of 36 kinds of immune cells among the samples in NOP58 high and low expression groups in the GSE68776 dataset. (**D**). Expression of PDCD1 between NOP58 high and low expression groups in the GSE17618 dataset. (**E**, **F**, **G**). The stroma, immune, and ESTIMATE scores between NOP58 high and low expression groups in the GSE68776 dataset. * means *P* < 0.05; ** means *P* < 0.01

Tumor immune microenvironment (TIME) plays a crucial role in the survival of tumor patients [36]. Hence, we explored the potential impact of NOP58 on immune cell infiltration in EwS. The EwS samples from the GSE17618 dataset was categorized into two groups according to the median expression value of NOP58: a high expression (high-NOP58) group and a low expression (low-NOP58) group. The infiltration proportions of 22 kinds of immune cells in each sample in the GSE17618 dataset were shown in Fig. 4A. Significantly, using the CIBERSORT algorithm, the infiltration proportion of M2 macrophages was reduced and the proportion of activated Mast cells was elevated in the high-NOP58 group, compared to the low-NOP58 group (Fig. 4B). Additionally, utilizing the GSE68776 dataset, we performed xCell to evaluate analysis immune cell infiltration between high- and low-NOP58 groups. The results showed that the levels of CD4 memory T cells, common lymphoid progenitors (CLPs), CD4 + T helper 2 cells (Th2 cells) were notably increased, but the levels of CD4 effector memory T cells, endothelial cells, cancer associated fibroblasts (CAFs), M1 macrophages and NK T cells were significantly reduced in the high-NOP58 groups, compared to the low-NOP58 group (Fig. 4C).

Subsequently, we analyzed the differential expression of six immune checkpoint genes PD-1 (PDCD1, programmed cell death 1), CTLA4 (cytotoxic T-lymphocyte associated protein 4), T cell immunoreceptor with Ig and ITIM domains (TIGIT), lymphocyte activating 3 (LAG3), interferon gamma (IFNG), and granzyme A (GZMA) between the high- and low-NOP58 groups using the GSE17618 dataset, and the results showed that the expression of PDCD1 in the high-NOP58 group was significantly higher than that in the low-NOP58 group (Fig. 4D). Furthermore, based on the data from the GSE68776 dataset, the levels of stroma, immune, and ESTIMATE scores were all remarkably elevated in the low-NOP58 group compared to the high-NOP58 group (Fig. 4E, F and G).

### Clinical value of NOP58 in EwS

**Fig. 5:**
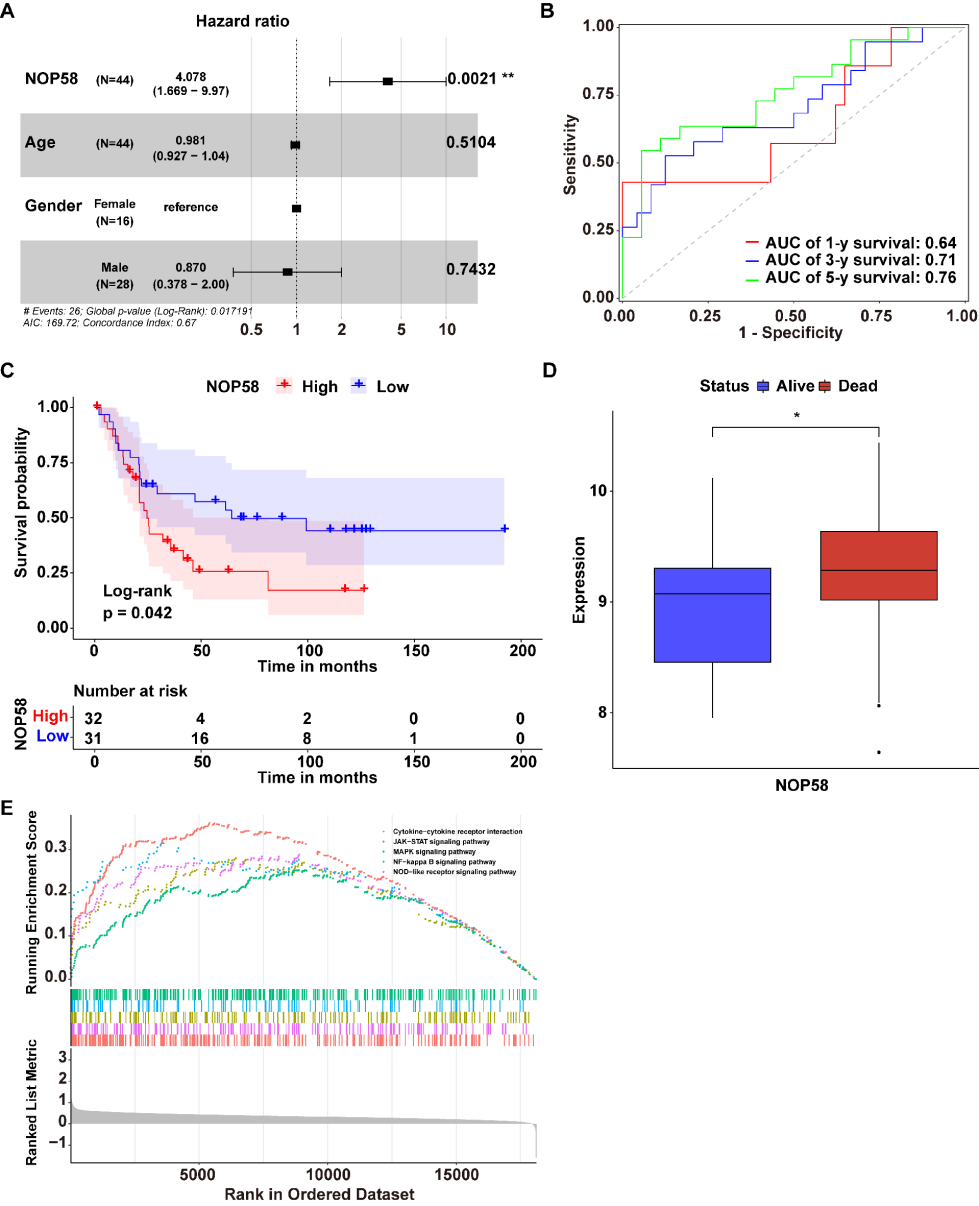
NOP58’s clinical value in EwS. (**A**). Results of multivariate COX regression analysis. (**B**). Time-dependent ROC curve of NOP58’s prognostic value at 1, 3 and 5 years. (**C**). Kaplan-Meier (KM) survival analysis based on GEO-meta datasets merged by GSE17618 and GSE218758 datasets. (**D**). NOP58 expression between Alive and Dead groups. (**E**) GSEA results. * meant p was smaller or equaled 0.05, ** meant p was smaller or equaled 0.01

Next, based on the data in the GSE17674 dataset, a multivariate Cox regression analysis was performed to describe the risk factors including NOP58 expression, age, and gender, showing that NOP58 was an independent prognostic predictor of EwS patients (Fig. 5A). The time-dependent ROC analysis demonstrated that the area under the curve (AUC) for the 1-year, 3-year, and 5-year survival were 0.64, 0.71, and 0.76, respectively, indicating that NOP58 may serve as a potential prognostic marker for EwS patients (Fig. 5B).

To investigate the relationship between NOP58 and the overall survival rates in EwS patients, we integrated two datasets (GSE17618 and GSE218758) into a merged set after removing the batch effects. According to the median value of NOP58 expression, EwS samples were divided into high-NOP58 and low-NOP58 groups, and Kaplan-Meier (KM) survival analysis was then performed. The results showed that patients in the high-NOP58 group had a relatively poor prognosis (Fig. 5C). Moreover, NOP58 expression levels were significantly differently between the Alive and the Dead groups, with the Dead group exhibiting markedly higher NOP58 expression compared to the Alive group (Fig. 5D).

### Pathways in EwS affected by NOP58

Using the EwS samples from the GSE68776 dataset, the samples were categorized into two groups (high- and low NOP58 groups) according to the median expression levels of NOP58. Subsequently, GSEA was conducted to explore the signaling pathways between two groups. The results revealed that 63 pathways were significantly enriched between two groups (Table S4). Among these pathways, five tumor development-related pathways including cytokine-cytokine receptor interaction, JAK-STAT signaling pathway, MAPK signaling pathway, NF-kappa B signaling pathway and NOD-like receptor signaling pathway, were notably activated in the high-NOP58 group, compared to the low-NOP58 group (Fig. 5E).

### Knockdown of NOP58 influenced the proliferation, invasion, apoptosis, and tumor growth of EwS in vitro and in vivo

**Fig. 6:**
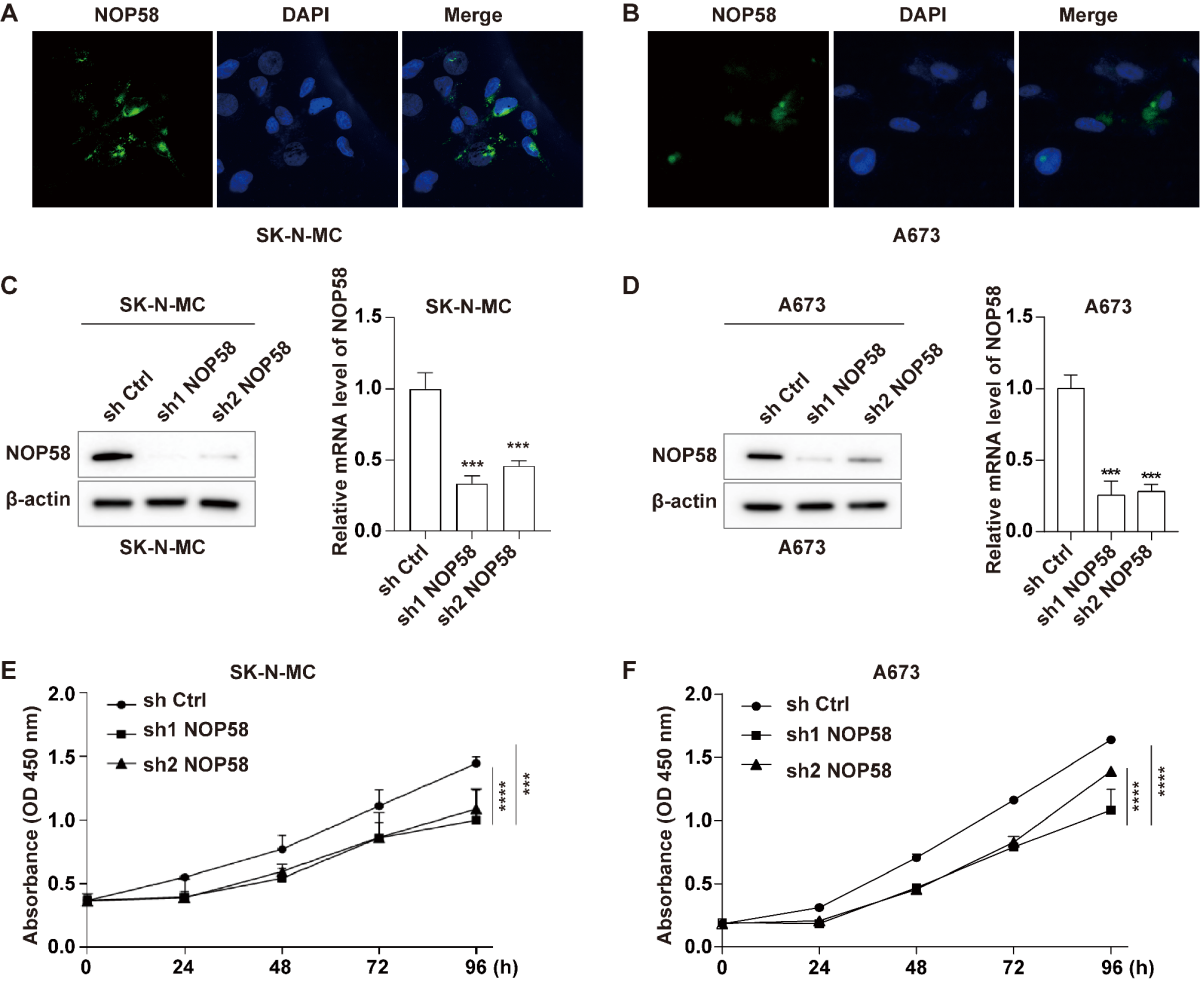
Knockdown of NOP58 influenced the proliferation of EwS cells. (**A**, **B**). An immunofluorescence assay was performed to detect the localization of the NOP58 protein in SK-N-MC and A673 cells using microscopy. (**C**, **D**). SK-N-MC and A673 cells were transfected with sh Ctrl, sh1 NOP58 and sh2 NOP58. Western blot and qRT-PCR assays were conducted to evaluate NOP58 expression in transfected cells. (**E**, **F**). CCK-8 assay was performed to assess cell proliferation. *** meant p was smaller or equaled 0.001; **** meant p was smaller or equaled 0.0001

NOP58 is a core component of box C/D small nucleolar ribonucleoproteins, which is involved in various cell physiological processes [37]. Firstly, we performed IF staining to validate the localization of NOP58 protein in EwS cells, including SK-N-MC and A673 cells. As shown in Fig. 6A and B, NOP58 protein was found to localize to the nucleolus and perinuclear region of SK-N-MC and A673 cells.

**Fig. 7:**
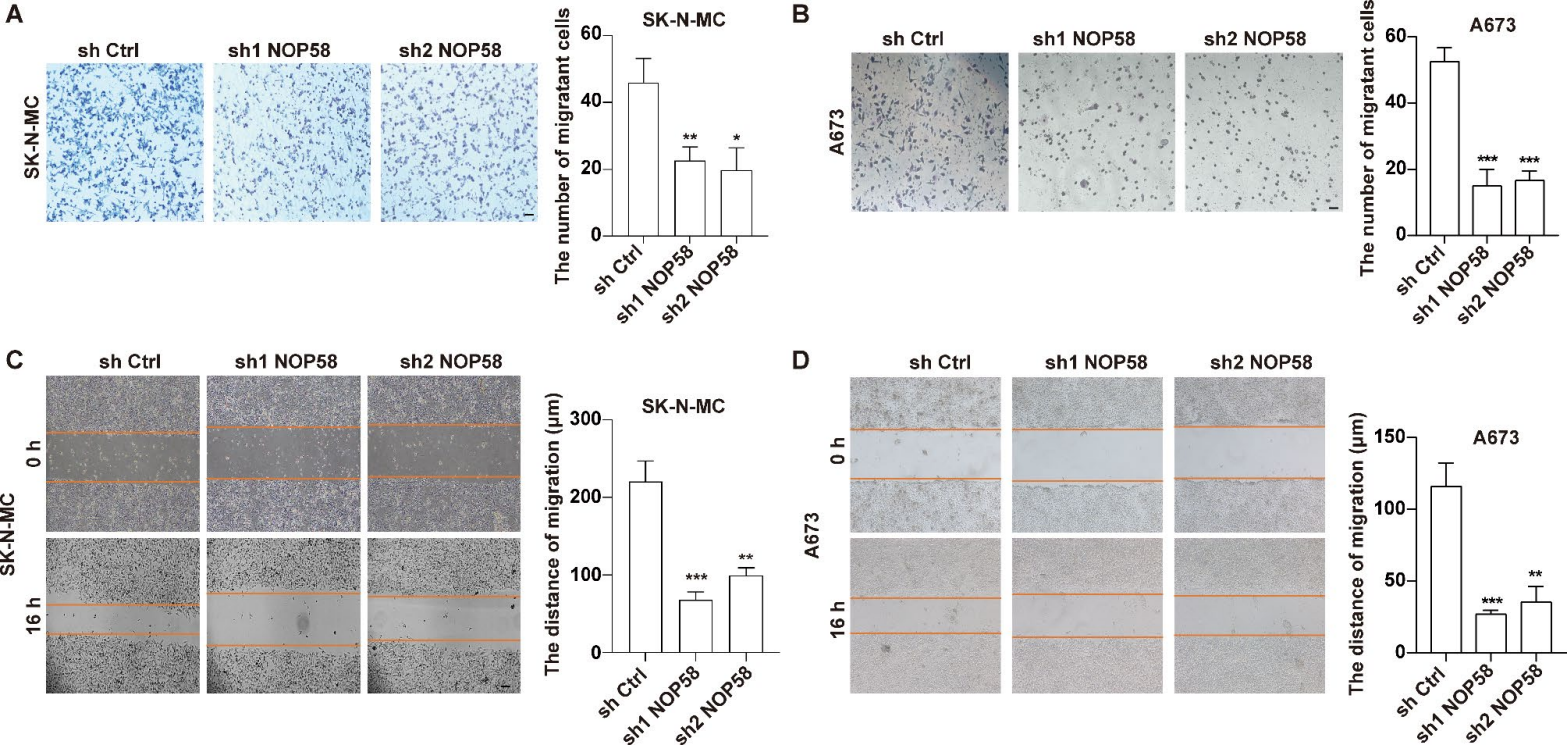
Knockdown of NOP58 influenced the migration of EwS cells. SK-N-MC and A673 cells were transfected with sh Ctrl, sh1 NOP58 and sh2 NOP58. (**A**, **B**). Transwell migration and (**C**, **D**) wound healing assays were utilized to determine cell migration. * meant p was smaller or equaled 0.05, ** meant p was smaller or equaled 0.01, *** meant p was smaller or equaled 0.001

Furthermore, to investigate the impact of NOP58 on EwS cells, we used NOP58 shRNAs to stably downregulate the expression of NOP58 in SK-N-MC and A673 cells. Western blot and qRT-PCR results indicated that both sh1 NOP58 and sh2 NOP58 significantly reduced NOP58 expression in SK-N-MC and A673 cells, compared to sh Ctrl (Fig. 6C and D). CCK-8 assay results demonstrated that downregulation of NOP58 significantly inhibited the proliferation of SK-N-MC and A673 cells compared to the sh Ctrl group (Fig. 6E and F). Transwell and wound healing assay results revealed that downregulation of NOP58 could suppress the migration of SK-N-MC and A673 cells compared to sh Ctrl (Fig. 7A, B, C and D).

**Fig. 8:**
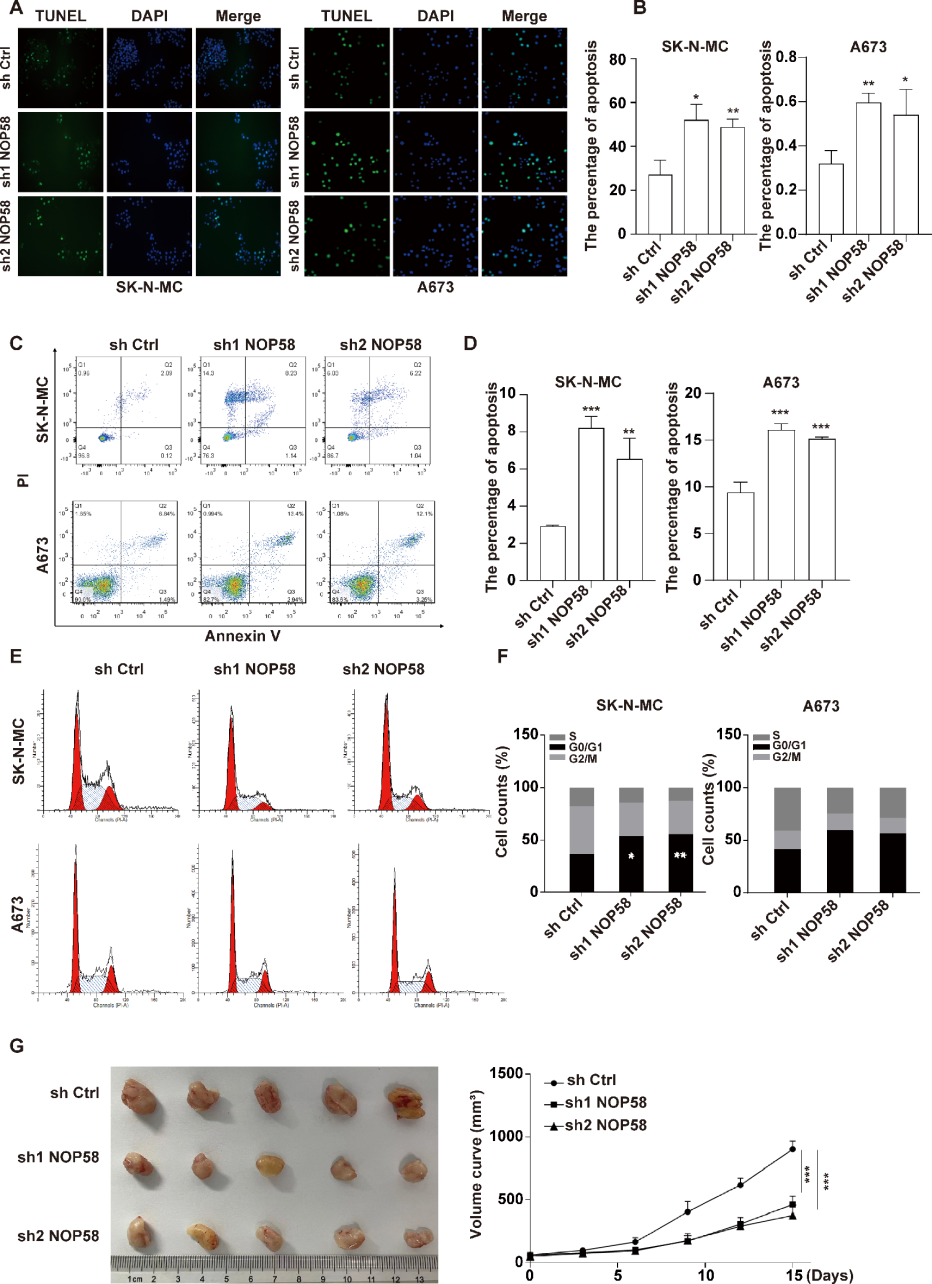
Knockdown of NOP58 influenced the apoptosis of EwS cells in vitro and tumor growth in vivo. (**A**, **B**, **C**, **D**). SK-N-MC and A673 cells were transfected with sh Ctrl, sh1 NOP58 and sh2 NOP58. Cell apoptosis was detected by TUNEL staining assay and flow cytometry. (**E**, **F**). Cell cycle distribution was determined by flow cytometry. (**G**). In vivo tumor growth of mice injected subcutaneously with NOP58-knockdown SK-N-MC cells. * meant p was smaller or equaled 0.05, ** meant p was smaller or equaled 0.01, *** meant p was smaller or equaled 0.001

Additionally, TUNEL assay was applied to assess whether NOP58 downregulation could affect apoptosis in EwS cells. It was observed that downregulation of NOP58 notably increased the number of TUNEL-positive cells in SK-N-MC and A673 cells compared to the sh Ctrl group (Fig. 8A and B). Subsequently, flow cytometry was used to assess the effect of NOP58 on the cell apoptosis and cell cycle progression. The results indicated that deficiency of NOP58 promoted apoptosis in SK-N-MC and A673 cells compared to sh Ctrl (Fig. 8C and D). Additionally, NOP58 deficiency led to a significant increase in the proportion of cells in the G0/G1 phase, while the proportion of cells in the G2/M phase decreased significantly compared to sh Ctrl (Fig. 8E and F), suggesting that NOP58 downregulation could induce cell cycle arrest.

Furthermore, to further explore the effect of NOP58 on tumor growth in vivo, we performed animal experiments using a SK-N-MC xenograft tumor mouse model. As shown in Fig. 8G, the tumor size of mice in the sh1 NOP58 and sh2 NOP58 groups was significantly reduced compared to the sh Ctrl group. These results showed that knockdown of NOP58 could inhibit tumor growth of EwS cells in vitro and in vivo.

## Discussion

In this study, we employed bioinformatics methodologies to identify the prognostic-related RBP gene in EwS [38, 39]. Evidence has demonstrated the close association between RBP genes and tumor prognosis. For instance, Liang et al. confirmed that Mex3a, a canonical RBP gene, is aberrantly overexpressed in lung adenocarcinoma, and its elevated expression level is significantly linked to poor patient prognosis [40]. Similarly, another study revealed that CMSS1, an RBP-related gene, is markedly upregulated in non-small cell lung cancer (NSCLC) tissues, with its high expression levels closely linked to unfavorable clinical outcomes in NSCLC patients [38]. These findings collectively highlight the potential role of RBP-related genes in prognostic evaluation. This research began with differential gene expression analysis comparing EwS with normal samples to identify differentially expressed RBPs. Subsequently, NOP58 was focused as the crucial RBP gene by PPI network analysis and univariate COX regression analysis. NOP58, also known as NOP5 or HSPC120 according to the gene database of National Center for Biotechnology Information (https://www.ncbi.nlm.nih.gov/gene/51602#summary), is primarily localized to the nucleolus [41]. It plays a crucial role in the biogenesis of the box C/D family of snoRNAs [42], which may serve as therapeutic targets or prognostic biomarkers of cancer [43, 44]. In this research, pan-cancer analysis revealed elevated levels of NOP58 across multiple cancer types, such as COAD, DLBCL and STAD. Furthermore, existing literature indicates that increased levels of NOP58 have been identified in various cancers, including prostate cancer, hepatocellular carcinoma and lung cancer, with its high expression linked to unfavorable outcomes of cancer patients [37, 44, 45]. The research by Jiang et al. indicated that overexpression of NOP58 was capable of facilitating lung cancer cell proliferation, migration and stemness [46]. Meanwhile, NOP58 has also been shown to influence chemoresistance in cancer [47]. The downregulation of NOP58 effectively diminished the chemoresistance of 5-fluorouracil (5-FU)-resistant colorectal cancer cells to 5-FU through inhibition of aerobic glycolysis [47]. In our study, we discovered for the first time that NOP58 expression was heightened in EwS tissues. Meanwhile, the increased expression of NOP58 was correlated with a poor prognosis for EwS patients, suggesting that it may serve as a prognostic marker in EwS. Furthermore, the knockdown of NOP58 was shown to suppress EwS cell proliferation and migration in vitro, and to prevent the tumor growth of EwS tumor-bearing mice in vivo. These findings also suggested that NOP58 may present a potential therapeutic target in EwS.

Next, to investigate the possible mechanisms by which this gene influences tumor progression, we conducted GSEA to pinpoint the cellular signaling pathways potentially linked to this gene. Our findings indicated that the high-NOP58 group exhibited activation of the JAK-STAT, MAPK, NF-kappa B and NOD-like receptor signaling pathways. These four pathways are known to be pivotal in the processe of tumorigenesis and cancer development [48–51]. Research has demonstrated that both the JAK-STAT3 and MAPK pathways are crucial for regulating tumor cell proliferation, apoptosis and stemness [52–54]. Furthermore, the STAT3 signaling is also linked to the tumor immune response, and plays significant roles in suppressing key immune activation regulators while facilitating the release of immunosuppressive factors [55]. Likewise, both the NF-kappa B and NOD-like receptor signalings not only impact tumor progression, but also contribute to chronic inflammation and tumor immune evasion with in the TIME [56, 57]. These results underscore their crucial involvement in tumor growth and the immune response regarding cancer. In this investigation, the results of the GSEA analysis suggested a potential relationship between NOP58 and the aforementioned four pathways. Thus, we hypothesized that NOP58 might play a role in the progression of EwS by affecting these pathways. Nonetheless, further studies are required to validate these hypotheses.

Evidence has demonstrated that NOP58, a core protein of box C/D small nucleolar ribonucleoprotein complexes, plays a critical role in 2’-O-ribose methylation [58]. 2’-O-ribose methylation is one of the most common modification essential to influence RNAs in multiple aspects, closely relating to human diseases [59]. Its patterns control the ribosomes’ intrinsic capabilities to translate mRNAs [60]. Box C/D snoRNA 89 can modify Bim through 2′-O-methylation and affect downstream signaling pathways to promote endometrial cancer [61]. Moreover, impairment of 2′-O-ribose methylation in 28 S rRNA inhibits hepatocellular carcinoma tumorigenesis [62]. In this study, the results of GO analysis presented in Table S3 showed that NOP58 is involved in the terms related to preribosome and sno(s)RNA-containing ribonucleoprotein complex. Hence, we infer that overexpression of NOP58 may promote the progression of EwS by modulating snoRNA-mediated 2’-O-methylation. However, these hypotheses require further exploration in future studies.

Information on immune landscape assists in elucidating what happens in the genesis of EwS and enhance the effect of immunotherapy. Wang et al. noted a positive correlation between NOP58 expression and the infiltration the levels of T helper type 2 (Th2) cells in hepatocellular carcinoma [37]. Research has demonstrated that Th2 cells exert pro-tumor role through the release of tumor-supporting factors IL4 and IL13 [63, 64]. Conversely, NKT cells and M1 macrophages are crucial for anti-tumor immunity [65]. In our research, we utilized Xcell algorithm to assess immune cell infiltration across high- and low-NOP58 groups. Our findings indicated an increase in CD4 effector memory T cells, M1 macrophages and NK T cells, in the low-NOP58 group when compared to the high-NOP58 group. On the other hand, the quantities of Th2 cells were significantly diminished in the low-NOP58 group. These results reveal that, in comparison to the high NOP58 group, the low NOP58 group exhibited elevated levels of certain anti-tumor immune cells while showing a notable reduction in pro-tumor Th2 cells. This suggests that NOP58 may affect tumor development by altering the infiltration of immune cells within the tumor microenvironment, and that reduced NOP58 expression may suppress tumor growth through the enhancement of anti-tumor immune reactions and the inhibition of pro-tumor Th2 cell infiltration. Nevertheless, further investigations are warranted to validate these findings and explore the underlying mechanisms.

Our study has several limitations. First, despite utilizing public databases such as GEO, the sample size for EwS specimens remains limited and needs to be increased. Additionally, the transcriptomic data sourced from the GEO database has predominantly been generated through Microarray or RNA sequencing methods. Although transcriptomic data offers significant insights into gene expression trends, the analysis is often impacted by both technical and biological biases [66], Consequently, it is essential to have additional validation with larger sample sizes to confirm the prognostic role of NOP58 in EwS and its association with immune cell infiltration. Second, it has been shown that liquid biopsy is an economical and minimally invasive substitute for conventional tissue biopsy when it comes to identifying tumor biomarkers in cancer patients [67, 68]. Common liquid biopsy analytes include cell-free DNA (cfDNA), RNA, microRNA (miRNA), and proteins [69]. In this research, we found that protein levels of NOP58 were significantly higher in EwS cells. However, it remains unclear whether NOP58 function as a biomarker for the early screening or diagnosis of EwS. Future research should explore the potential role of NOP58 in EwS diagnostics and monitoring using proteomics or liquid biopsy-based approaches. Chen et al. highlighted the growing significance of vaccine technology and nanomedicine in oncology in their review about the bioscience and medical fields in 2023 [70]. Given the demonstrated oncogenic role of NOP58 in EwS, future investigations could consider developing nanoparticle-based delivery systems for NOP58-targeting agents (e.g., siRNA or CRISPR). Integrating this targeted approach with existing therapies may enhance treatment efficacy and potentially improve outcomes for EwS patients with high NOP58 expression, warranting further preclinical and clinical exploration.

Collectively, in this study, NOP58 was found to significantly overexpressed in EwS samples compared to normal controls. The knockdown of NOP58 resulted in the inhibition of proliferation and migration of EwS cells in vitro, and a reduction in tumor size in vivo. Furthermore, NOP58 was determined to have prognostic significance, with high expression correlating to poor prognosis in EwS patients. These findings showed that NOP58 may serve as a potential therapeutic target and prognostic marker for EwS treatment.

## Electronic supplementary material

Below is the link to the electronic supplementary material.

Supplementary Material 1

Supplementary Material 2

Supplementary Material 3: Table S1. RBP genes used for candidate genes screening.

Supplementary Material 4: Table S2. 179 candidate genes.

Supplementary Material 5: Table S3. GO and KEGG enrichment analysis.

Supplementary Material 6: Table S4. GSEA analysis.

## Data Availability

The datasets (GSE68776, GSE45544, GSE17674, GSE17618, GSE17679, GSE218758) analyzed during the current study are openly available in the GEO (Gene Expression Omnibus, https://www.ncbi.nlm.nih.gov/geo/). https://dcc.icgc.org/

## Abbreviations

DEGs: Differentially expressed genes
EwS: Ewing sarcoma, or Ewing’s sarcoma
GSEA: Gene set enrichment analysis
hMSC: Human mesenchymal stem cells
MAPK: Mitogen-activated protein kinase
MEM: Minimum Essential Medium
MNC: Maximum neighborhood component
NEAA: Non-essential amino acids
RBPs: RNA-binding proteins
PDCD1: Programmed Cell Death Protein 1
ROC: Receiver operating characteristic
PPI: Protein-protein interaction
RNPs: Ribonucleoproteins
