# Supplementary figures and images for: RNA-binding protein gene NOP58 exhibits crucial prognostic and therapeutic value in Ewing sarcoma
Source: Hereditas. 2025 May 14;162:76. doi: 10.1186/s41065-025-00440-5 (PMC12076867; doi:10.1186/s41065-025-00440-5)

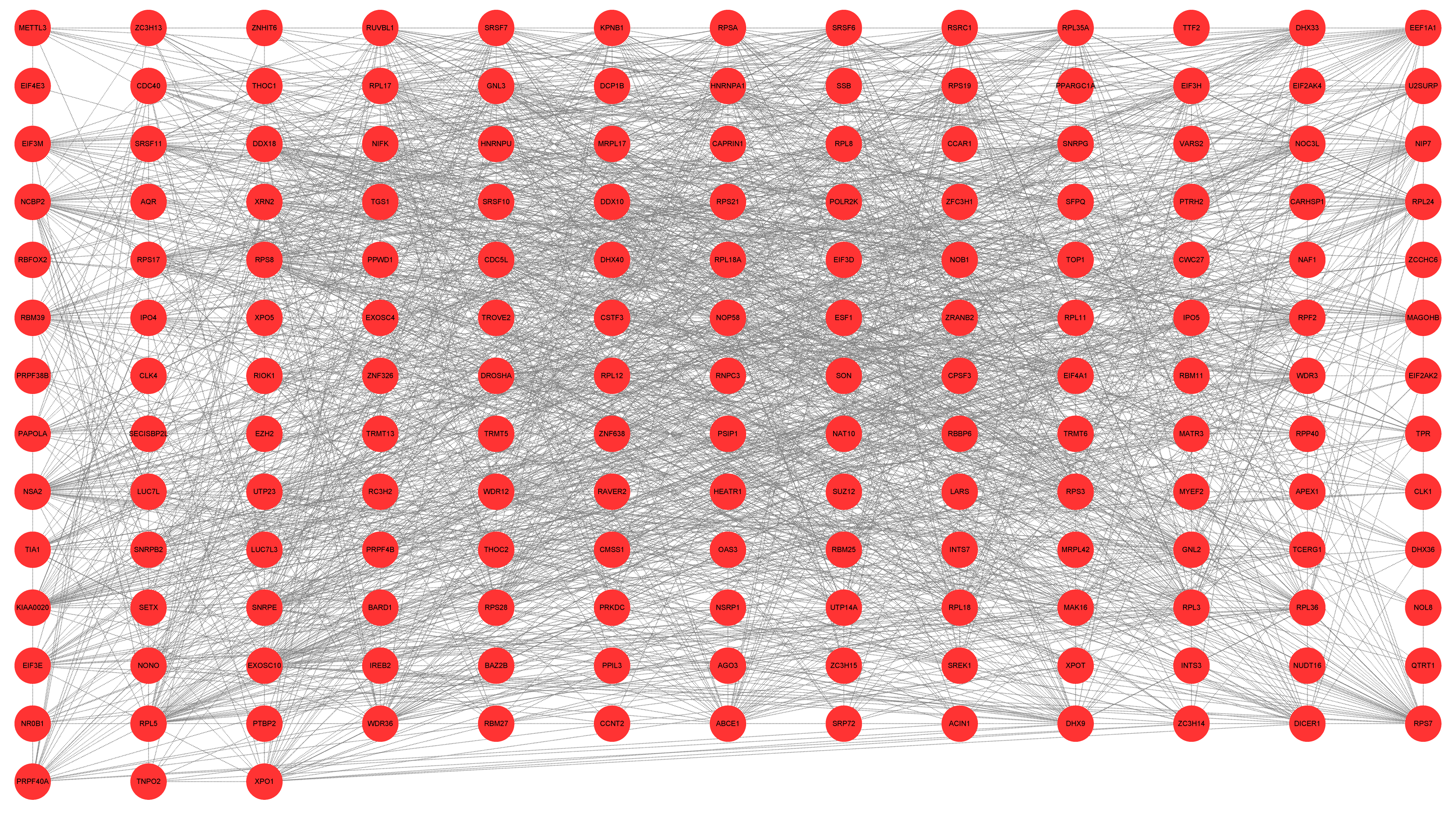

Supplement: Supplementary file 1 — Supplementary Material 1 [file 41065_2025_440_MOESM1_ESM.tif]

NOP58

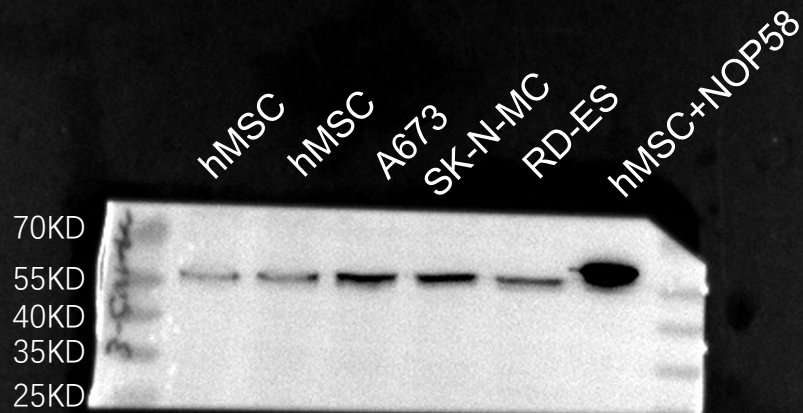

$\beta$ -actin

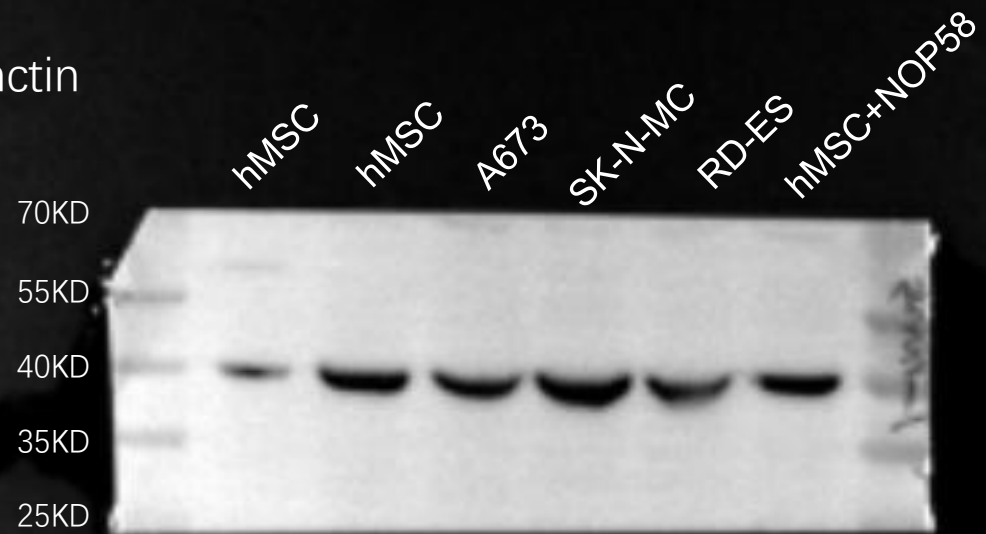

NOP58

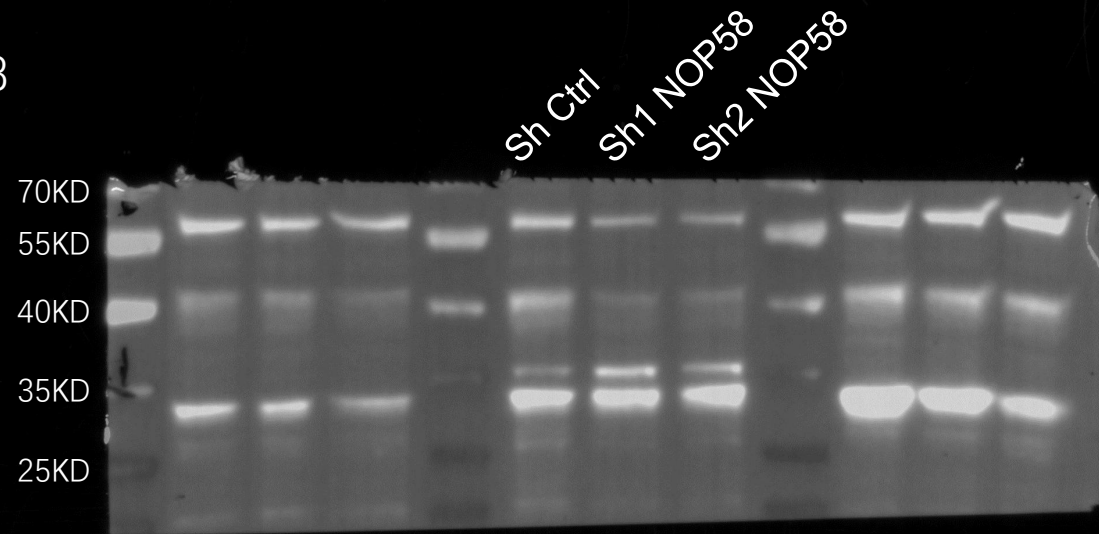

$\beta$ -actin

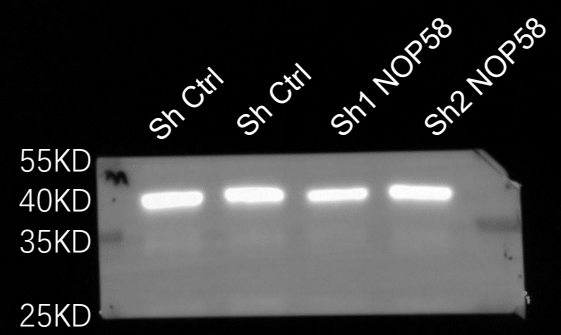

Supplement: Supplementary file 2 — Supplementary Material 2 [file 41065_2025_440_MOESM2_ESM.pdf]
